# What is the best core measure after critical illness when the IES-R is no longer accessible for new researchers?

**DOI:** 10.1186/s13054-019-2595-2

**Published:** 2019-09-13

**Authors:** Reba Umberger

**Affiliations:** Department of Acute and Tertiary Care, College of Nursing, The University of Tennessee Health Science Center, 920 Madison Avenue, Suite 564, Memphis, TN 38163 USA

Dear Dr. Vincent,

The recent publication by Hosey et al. regarding validation of the Impact of Event Scale-6 to screen for post-traumatic stress disorder (PTSD) in ARDS survivors is an important contribution for research on outcomes after critical illness [1]. PTSD is one possible manifestation of post-intensive care syndrome which may occur and impact long-term outcomes after critical illness [2]. Experts in long-term outcome by consensus have recommended the Impact of Event Scale-revised (IES-R) as one core outcome measure for mental health following critical illness [3]. Hosey et al. conducted a secondary analysis of 1001 ARDS survivors with IES-R measures at multiple points over 5 years and validated a shorter 6-question version [1]. The IES-6 includes 2 questions for each PTSD domain based on DSM-IV criteria (see Fig. 1). This shortened version will greatly reduce the screening burden on participants; however, the authors also recommend longitudinal administration of both the IES-R and the IES-6 after critical illness to address stability of the measure using test-retest reliability. Unfortunately, the IES-R scale has been retired by the developer due to revisions in PTSD criteria, limiting its use to investigators with ongoing research or prior permission (personal communication with Dr. Weiss, August 12, 2019). While the IES-R does not fully align with the DSM-V criteria, it aligns with three criteria. The PTSD Checklist for DSM-5 (PCL-5) has been recommended as a screening measure of PTSD and adopted by the National Center for PTSD. The PCL-5 aligns fully to DSM-V criteria and includes questions to assess for negative alterations in cognition and mood (e.g., blame, negative emotions) [4] which are not assessed by the IES-R or IES-6. The importance of this new PTSD symptom cluster is unknown after critical illness. Researchers should use the most reliable and valid instruments available at the time their research is designed. The PCL-5, a 20-question instrument, has not yet been validated in ICU populations [5]. More work will be needed in the future to validate the PCL-5 in ICU populations and to create and validate a shorter version to reduce the burden on participants.

**Fig. 1:**
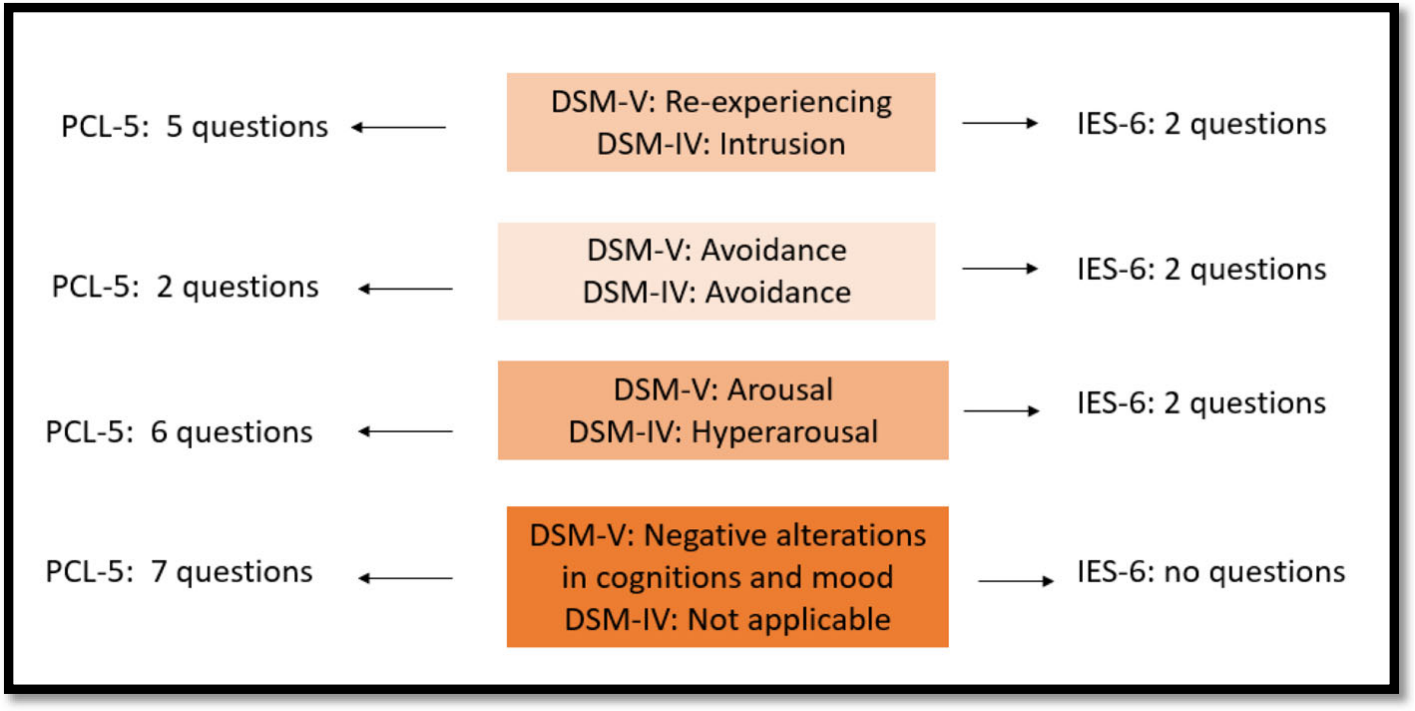
Comparison of PCL-5 and IES-6 questions addressing PTSD criteria. Note: Current DSM-V criteria are shown with analogous DSM-IV criteria which were used to develop the IES-R and hence the IES-6

`

Sincerely,

Reba Umberger, PhD, RN, CCRN-K

Assistant Professor of Nursing

University of Tennessee Health Science Center

Department of Acute and Tertiary Care

## Data Availability

Not applicable.
